# Correction to “Structural Properties of Hf_0.5_Zr_0.5_O_2_ Integrated on Silicon”

**DOI:** 10.1021/acsaelm.5c02645

**Published:** 2026-01-07

**Authors:** Kit de Hond, Mart Salverda, Majid Ahmadi, Evert Houwman, Beatriz Noheda, Bart J. Kooi, Guus Rijnders, Gertjan Koster

The original version of this article points to a wrong file as Supporting Information. This was corrected.

## Supplementary Material



## Notes

Contains figures showing: The Supporting Information is available free of charge at https://pubs.acs.org/doi/10.1021/acsaelm.5c02645.

X-ray reflectivity data and X-ray diffraction ϕ-scans (PDF)

