# Supplementary material for: Correction to “Structural Properties of Hf0.5Zr0.5O2 Integrated on Silicon”
Source: ACS Appl Electron Mater. 2026 Jan 7;8(2):1026. doi: 10.1021/acsaelm.5c02645 (PMC12854731; doi:10.1021/acsaelm.5c02645)
Supplement: Supplementary file 1 [file el5c02645_si_001.pdf]

Supporting information: Structural properties of $Hf_{0.5}Zr_{0.5}O_2$ integrated on silicon

Kit de Hond[1], Mart Salverda[2], Majid Ahmadi[2], Evert Houwman[1], Beatriz Noheda[2], Bart J. Kooi[2], Guus Rijnders[1], Gertjan Koster[1,a)]

[1]MESA+ Institute for Nanotechnology, University of Twente, P.O. Box 217, 7500 AE Enschede, the Netherlands

[2]Zernike Institute for Advanced Materials, University of Groningen, Groningen, the Netherlands

[a)] Electronic mail: g.koster@utwente.nl

Figure S1 shows x-ray reflectivity (XRR) measurements for samples with different HZO thicknesses. The measured scans are fitted by X'Pert Reflectivity software from Malvern Panalytical. For the fitting, the STO layer is set to 4 nm, the LSMO to 20 nm and the HZO to the expected thickness. The final fit is obtained by utilizing the piecewise optimization algorithm of the software. During this optimization the parameters are kept within certain boundaries (±15% of expected values).

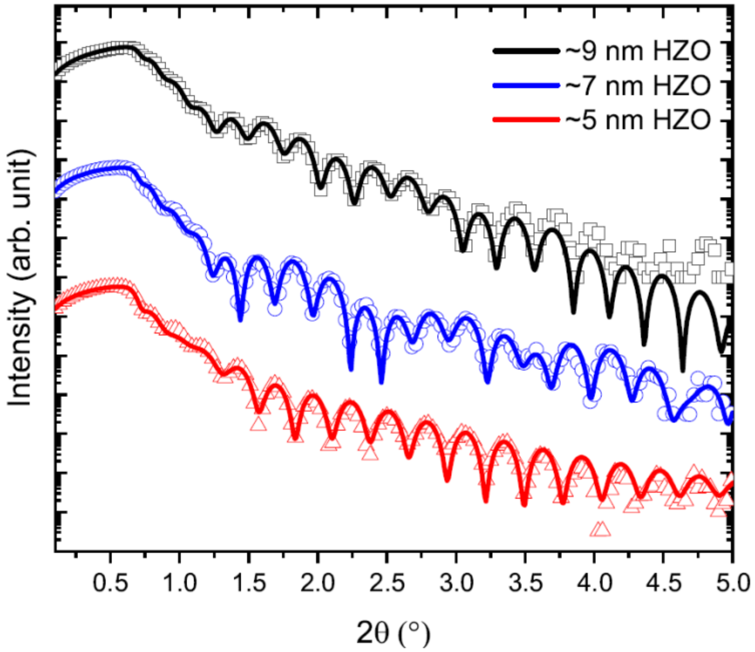

Figure S1: XRR measurements of samples with different HZO thicknesses. The symbols are the experimental data and the lines correspond to the simulated patterns.

Figure S2 shows φ-scans of the LSMO and silicon (202) reflections. For both materials the four peaks separated by 90° originate from the cubic symmetry. The layers show an in-plane rotation of 45° with respect to each other, From this we can conclude that the epitaxial relationship between the silicon and the STO/LSMO is as follows: LSMO $(001)_{pc}$//Si (001) and LSMO $(100)_{pc}$//Si (110).

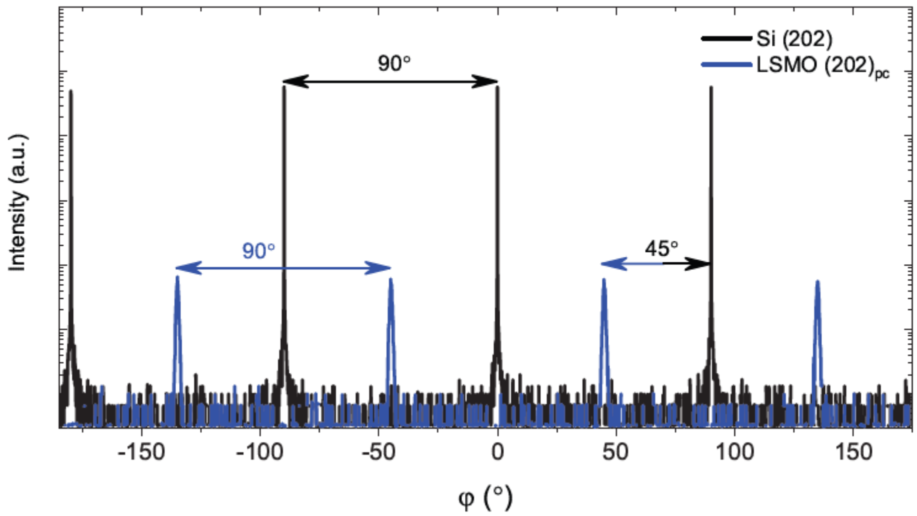

Figure S2: φ-scans of the LSMO and silicon (202) reflections. The *pc* subscript denotes the pseudo cubic orientation of the LSMO crystal.
